# Relationship between malaria, anaemia, nutritional and socio-economic status amongst under-ten children, in the North Region of Cameroon: A cross-sectional assessment

**DOI:** 10.1371/journal.pone.0218442

**Published:** 2019-06-21

**Authors:** Nobelle Sakwe, Jude Bigoga, Judith Ngondi, Boris Njeambosay, Livo Esemu, Célestin Kouambeng, Philomena Nyonglema, Clovis Seumen, Inocent Gouado, Julius Oben

**Affiliations:** 1 Laboratory of Nutrition and Nutritional Biochemistry, Department of Biochemistry, University of Yaounde I, Yaounde, Cameroon; 2 Molecular Parasitology and Disease Vector Research Laboratory, the Biotechnology Center, University of Yaounde I, Yaounde, Cameroon; 3 National Malaria Control Programme, Yaounde, Cameroon; 4 Laboratory of Immunology, University of Yaounde I, Yaounde, Cameroon; 5 Laboratory of Nutrition and Food Sciences, Department of Biochemistry, University of Douala, Douala, Cameroon; University of Dhaka, BANGLADESH

## Abstract

**Background:**

Despite malaria, malnutrition and anaemia being major public-health challenges in Cameroon, very little has been reported on the interaction between these interconnected health determinants. This study therefore sought to investigate the relationship between malaria, anaemia, nutritional and socio-economic status amongst under—ten children living in six localities within two health districts in the North Region of Cameroon.

**Methods:**

Accordingly, a cross- sectional survey was conducted during the peak malaria season in November 2014, in Pitoa and Mayo-Oulo Health Districts. Three hundred and sixty eight children aged 6months—10 years were enrolled. Structured questionnaires were used to assess socio-economic status. Anthropometric indices were taken using standard methods and nutritional status assessed by calculating Height for Age (HA), Weight for Age (WA) and Weight for Height (WH) z-scores to determine stunting, underweight and wasting respectively. Finger-prick blood samples were used to prepare thin and thick blood smears for microscopy. Whole blood was collected to determine the PCV and blood spots on filter paper were used to extract plasmodium DNA for speciation by PCR.

**Results:**

Overall prevalence rates of malaria, malnutrition and anaemia were 32.9%, 54.1% and 20.6% respectively. Stunting, underweight and wasting were detected in 56.9%, 63.5% and 34.8% of the children respectively. There was a significant association between malaria and malnutrition [OR = 1.89, (95% CI: 1.12–3.19); (p = 0.017)]. Malnutrition was also strongly associated with malaria status [OR = 2.07, (95% CI: 1.22–3.53); (p = 0.007)]. The prevalence rates of mild, moderate and severe anaemia were 8.1%, 9.2% and 3.3% respectively. Both malaria status and anaemia correlated with development index [OR = 0.75, (95% CI: 0.58–0.99); (p = 0.042)] and [OR = 1.45, (95% CI: 1.05–2.00); (p = 0.023)] respectively.

**Conclusion:**

Our findings show a synergistic relationship between malaria and malnutrition. Effective collaboration between malaria control and nutrition intervention programmes is essential for proper case management and improved socio-economic status.

## Introduction

Malaria, anaemia, and malnutrition are public-health challenges in paediatric populations in sub-Saharan Africa with malaria and undernutrition being major contributors to childhood mortality [1]. Malaria is a serious vector borne disease affecting mainly impoverished communities. Although children are usually the most vulnerable, the disease can be worsened in those that are malnourished [2, 3]. Despite efforts by control programmes to reduce the disease burden, it still places the lives of many in jeopardy, especially in sub-Saharan Africa, where over 90% of the deaths occur, 78% of which are in children less than 5 years old [4–6]. Recent data show that an estimated 435.000 people died of malaria in 2017, with 219 million cases of malaria reported in 90 countries [7]. In Cameroon, malaria is responsible for 34% of consultations and 47% hospitalization, 21% hospital related deaths of which 38% are in children less than 5years [8]. Previous reports in the same study sight revealed prevalence rates of malaria, malnutrition and anaemia to be 39%, 40.2% and 68.2% respectively [9].

On the other hand, undernutrition is known to cause more than 50% of deaths among under-five children [10]. It is also responsible for most premature mortalities in developing countries that are plagued by poverty and ignorance [11]. Although the World Health Assembly emphasises on the need for all children to be free of malnutrition in all its forms, and stunting rates are dropping, about 159 million around the world are still affected, the number of overweight children is increasing alarmingly and wasting still threatens the lives of 50 million children across the globe [12]. In Cameroon, about 52.000 children reportedly die of malnutrition annually with four of the regions (East, Adamawa, North, Far North) being the most affected [13].

Studies highlighting the relationship between malnutrition and malaria have so far been controversial and complex. While some believe that malaria may cause malnutrition, others observed that malnutrition might enhance susceptibility to malaria [14]. In heavily endemic areas, it is almost inevitable that malaria infection will be associated with anaemia, although it may not be the prime cause of it [15, 16]. Anaemia, an indicator of both poor nutrition and poor health is a common and sometimes serious complication of *P*. *falciparum* infection [17, 18]. This is frequent in developing countries where its causes are multi-factorial. Anaemia impairs normal development in children and it constitutes a major public health problem in young children in the developing world with wide social and economic implications [19], Cameroon inclusive.

Several studies [1, 5, 14, 20, 21, 22], have investigated the problem of malaria related to anaemia and nutritional status in children, in different regions of the world, most of whose findings have been inconsistent [23]. In Cameroon, very little information exists on the interaction between these interconnected health determinants especially in the northern parts that bear the highest burden. Thus this study aimed at contributing to a better understanding of the relationship between malaria, malnutrition, anaemia and socio-economic status amongst children (0-10years) in 2 Health districts, (Pitoa and Mayo- Oulo) in the North Region of Cameroon. It will provide baseline information for future studies that will guide better management and control where both malaria and malnutrition prevail.

## Materials and methods

### Study area and study population

The study was conducted in two health districts, namely, Pitoa Health District, 9.21°N, 13.31°E and Mayo-Oulo Health District, 9.46°N, 13.44°E, and both peri-urban settings, in the north region of Cameroon. The area is the center of trade of the surrounding agricultural region and also houses several textile processing facilities. Pitoa has a population of about 108.611 inhabitants while Mayo-Oulo is inhabited by about 91.501 individuals. The population depends almost entirely on agriculture (including gardening) for subsistence. Crops grown include; rice, maize, millet, cotton, and sorghum for Pitoa whereas beans peanuts and maize are cultivated in Mayo-oulo. The study area is predominantly populated by individuals of the Hausa ethnic group. Polygamy is a common practice with a number of wives and their children living in separate huts within the family compound. Both health districts have a Sudanese type climate with an annual average rainfall of 700–1000mm, annual average temperature of 26.0–33.0°C and relative humidity of 15%. Malaria is endemic in the region, with a seasonal mode of transmission, lasting about 6 months and peaking generally during the months of September—November when rainfall is highest. *Plasmodium falciparum* is the predominant (>98%) parasite species in the area.

The study population consisted of children aged 6months to 10 years and of both sexes, after full informed consent. They weighed between 4kg and 30.8 kg. Children with recent (during the last 48hours) or current fever, were considered symptomatic, and were tested using Rapid Diagnostic Tests and immediately treated for free, following the national guidelines for managing uncomplicated malaria, if found positive. Blood smears were taken for all children irrespective of fever status for microscopy.

### Study design and sampling procedures

This was a cross-sectional study, carried out in November 2014, during the peak malaria transmission season. It was conducted as part of a larger study to determine the impact of insecticide resistance on Long Lasting Insecticidal Nets (LLIN) and malaria burden in the North of Cameroon.

Initially, a census of all households in the study localities was conducted and all houses given unique survey numbers. Subjects in each household were registered and information including name, sex, age, relationship to the head of the household, demographic and socio- economic data as well as risk factors like, marital status, caregiver level of education, employment status of family head, type of housing, availability and principal source of electricity, drinking water and type of toilets at home, possession of household equipment and participation in social groups, were recorded.

The head of each household was the principal respondent and provided all necessary information for the household. Following completion of the census, the study participants were recruited from 58 households through systematic random sampling. Visits to households were in the afternoon to include children attending school. The investigation methods included the use of structured questionnaires, clinical evaluation and laboratory investigations.

### Ethical considerations

The study was carried out as part of a larger project to assess the Impact of Insecticide Resistance on the effectiveness of LLINS and malaria burden in the North of Cameroon, for which an ethical clearance was obtained from the National Ethics Committee of Cameroon (No. 102/CNE/SE/09; FWA IRB00001954). Participation in the study was strictly voluntary with written informed consent from legal parents/guardian.

### Field procedures- data collection

#### Structured questionnaire

A Structured questionnaire was used for data collection. Certified Nurses, Medical Laboratory Technicians and Interviewers were trained for data and sample collection. The Questionnaires were administered following an informed consent process, to record socio-economic and demographic data such as sex of the child, age of the child, educational status, occupation as well as medical history as concerns malaria, questions on malaria prevention practice like use of LLIN, nutrition /feeding habits and anthropometric measurements were included for each household. Weighing scales were calibrated daily using 5kg weight. All data collection instruments were pretested and validated.

#### Clinical evaluation and anthropometric measurements

Clinical evaluations were carried out by trained medical personnel (Nurses). Axillary body temperature of the children were taken using digital thermometers and recorded in °C (+0.5°C). The conjunctivas were also examined for paleness (signs of anaemia). Conjunctival pallor was evaluated by everting the lower eyelid and examining the palpebral conjunctiva. This was recorded as either coloured or not coloured. A child was considered febrile if he/she had temperature ≥ 37.5°C or had reported they had fever during the past 48 hours.

With regards to nutritional status, measurements were performed according to standard anthropometric methods and related to age and sex [24]. This was carried out by 2 trained data collectors/interviewers with at least University education, hailing from the region with a good mastery of the local vernacular and French. The children were weighed wearing light clothes only and no shoes by using a portable digital scale accurate to 0.1 kg. In the case of infants (˂ 2 years), the weight of the mother was recorded and subtracted from the weight of the mother plus the infant, to get the weight of the infant. Length (for children aged ˂ 2 years), and height (for children >2 years of age) were measured to the nearest 0.1cm using a non-stretchable tape. Height was measured in a standing position while Length was measured using the same tape, with the child in a horizontal position.

#### Nutritional status assessment

As indicators of nutritional status, Z-scores for weight-for-height (WH), weight-for-age (WA) and height-for-age (HA) were used. In order to analyse the nutritional status, the Weight and Height of the boys and girls were compared to those of same aged boys and girls measured in the National Centre for Health Statistics (NCHS)/WHO growth reference curves using the nutrition module of the Epi Info 2000 programme. Children were classified as stunted, underweight, or wasted if their HAZ, WAZ, or WHZ was < −2, respectively. While Z-scores < -3.0 was used to classify severely stunted, severely underweight and severely wasted children. The WHO Classification for assessing severity of malnutrition by percentage prevalence ranges of these indicators among children was followed. Children with < −2 and < −3 SD were classified as malnourished and severely malnourished respectively.

#### Socio-economic status (SES) assessment

To determine the SES of the households, the development index (a composite variable) was computed using socio-economic indicators. Indicators for socio-economic status included; the number of people living in a household, the level of education of caregiver and family head, employment status of family head, type of housing, type of toilet, source of lighting and cooking energy, availability of potable water and possession of household assets.

All the socio-economic indicators were recoded in ranking order of importance and the sum of scores calculated for each household. A straight forward count of the possessions was made where one point was given for each of the possessions to obtain the development index. The index was then divided into quintiles, SES 1 to SES 5 to determine poorest, poor, average, rich and richest SES as seen below (Table 1).

**Table 1:** Assessment of socio- economic status.

SES	Quintile
Richest (SES 5)	Very high level
Rich (SES 4)	High level
Average (SES 3)	Medium level
Poor (SES 2)	Low level
Poorest (SES 1)	Very low level.

#### Blood collection and processing

All febrile cases (temperature ≥ 37.5 °C) or cases with history of fever (during the past 48 hours) were tested with an RDT (SD- Bioline, Standard Diagnostics, INC. Lot No. 05FK60) using finger prick blood samples, and treatment given, if found positive. Still, finger prick blood (one to two drops) was immediately dispensed on properly labelled slides and used to prepare thin and thick blood smears from all participants (irrespective of fever status) for microscopy; blot filter paper (Whatman 3MM) [25] for parasite DNA isolation and species identification by polymerase chain reaction (PCR) and for the determination of the packed cell volume (PCV).

#### Malaria microscopy

The thick and thin blood smears were air-dried, fixed with methanol (thin smears only) and then stained with Giemsa stain and microscopically examined for the presence of malaria parasite following standard protocol [26]. Each slide was examined by two independent microscopists blinded of each other’s results. Parasite density was determined by counting the number of parasites present per 200 white blood cells (WBC) in a thick smear and multiplying by 40 to arrive at an approximate parasite count per microliter of blood. This was based on the assumption that the average WBC count was 8.000/uL blood. The thin smears were used to identify the *Plasmodium* species [26].

#### PCR identification of *Plasmodium* species

Plasmodium species identification was further confirmed by PCR, using *Plasmodium* DNA extracted by the Chelex Method from a portion of each blood sample collected on filter paper [25, 27].

#### Determination of the Packed Cell Volume

The PCV was determined by microcentrifugation of the blood filled microcapillary tubes. Anaemia in children was defined as a PCV less than 33% and/or Hb level less than 11g/dl. However, this was further categorized as either mild (Hb between 10.1 and 10.9 g/dL), moderate (Hb between 7.0 and 10.0 g/dL) and severe (Hb < 7.0 g/dL).

### Definitions and end-points

Fever was defined as axillary temperature ≥ 37.5°C. Malaria parasitaemia was defined as any asexual parasitaemia detected on a thick or thin blood smear. Confirmed malaria (CM) was defined as the presence of any species of *plasmodium*, with an axillary temperature of ≥ 37.5°C or reported fever in previous 48 hours. The term “parasitaemia” refers to a positive result on expert microscopy. A Haemoglobin level of < 11g/dL was classified as anaemic and further categorized as mild (Hb between 10.1 and 10.9 g/dL), moderate (Hb between 7.0 and 10.0 g/dL) and severe (Hb< 7.0 g/dL). Height–for- age (HAZ), Weight- for-age (WAZ) and Weight-for- height (WHZ) Z-scores were calculated from Center for Disease Control (National Center for Health Statistics)/ World Health Organization (2006) reference values using Epi Info 2000 software (version 2000, Atlanta, GA). Children were classified as stunted, underweight or wasted if the HAZ, WAZ and WHZ was < -2 SD of the NCHS/WHO reference median, respectively. They were classified as having severe stunting, wasting or underweight, if the HAZ, WHZ, or WAZ was <-3, respectively.

### Statistical analyses

All data collected were entered in Microsoft Excel 2010 and statistically analyzed using the Statistical Package for Social Sciences (SPSS) Standard version, Release 20.0 (SPSS Inc. 2012). Scale (continuous variables) as parasitaemia and Z-scores were described using measurements of central tendencies (Geometric Mean, Median) and measurements of dispersion (Standard Deviation and range while highlighting the minimum and the maximum values). The scale variables were screened for normality using Kolmogorov-Smirnov and Shapiro-Wilk tests for normality as to decide whether parametric or non-parametric tests should be used for analysis. The variables generally significantly departed from the theoretical normal distribution (P<0.05) and non-parametric tests were then used to compare groups for significant differences notably Man-Whitney U test to compare two independent samples.

For the categorical variables such as gender, anaemic status, malaria status etc. they were described using frequencies and proportions. Bivariate association between predictors of malnutrition, anaemia and malaria was computed using the Crammer’s V test. Logistic Regression Model (LRM) was used to depict significant predictors or risk factors for malaria, malnutrition and anaemia using the Wald Statistics to generate the significance level and the Odd Ratios (OR) and the 95% CI of ORs. The method used was the SPSS standard regression analysis ‘Enter’.

## Results

### Characteristics of the study population

A total of 368 children residing in the north region of Cameroon, of both sexes, males 175 (48.3%), females 187 (51.7%), aged ≤10 years old with a mean age of 4.7years ± 2.5 (6months to 10years), mean weight of 14.9kg ± 5.5 (4.0 kg to 30.8kg) and mean height of 98.53cm ± 22.08 (27cm to 108cm) were screened for the presence of malaria parasite and anaemia as well as nutritional and socio-economic status assessed. Out of the 368, 362 had complete assessment data. The proportion of children aged ˂5 years was 182 (50.3%) almost equal to that of those aged 5 years and above 180 (49.7%). The mean ± SD of HAZ, WAZ and WHZ scores were −1.621±2.898, −1.462±1.986 and -0.269±2.547 respectively in the ˂5 age group while for the ≥5 age group it was −1.346±2.315, −1.503±1.258 and -0.520±2.610 respectively. (Table 2).

**Table 2:** Baseline characteristics of the study population.

	Parameter			All
Age group		˂ 5	≥ 5	Total
**Sex**	**Female n (%)**	85(45.5%)	102(54.5%)	187(51.7%)
	**Male n (%)**	97(55.4%)	78(44.6%)	175(48.3%)
	**Mean WAZ**	-1.462±1.986	-1.503±1.258	-1.489±1.622
	**Mean HAZ**	-1.621±2.898	-1.346±2.315	-1.484±2.607
	**Mean WHZ**	-0.269±2.547	-0.520±2.610	-0.395±2.578
	**Children by age group n (%)**	182(50.3%)	180(49.7%)	362(100%)
	**Parasitaemia n (%)**	86(23.8%)	33(9.1%)	119(32.9%)
	**Geometric Mean Parasite Density (range)**	2224±23008 (40–129600)	1300±10318 (80–69200)	1783 ±19118 (40–129600)
	**Confirmed Malaria (RDT) n (%)**	52(89.7%)	39(88.6%)	91(89.2%)
	**Malnutrition n (%)**	101(55.5%)	95(52.8%)	196(54.1%)
	**Stunting n (%)**	104(57.1%)	102(56.6%)	206(56.9%)
	**Underweight n (%)**	106(58.2%)	124(68.9%)	230(63.5%)
	**Wasting n (%)**	57(31.3%)	69(38.3%)	126(34.8%)
	**Anaemia**	40(22.2%)	34(19.0%)	74(20.6%)

#### Prevalence of malaria

A total of 362 samples were collected from 6 villages (Kirambo, Guizigaré and Boussa in Pitoa Health District; and Mayo- Oulo, Bala and Dourbeye in Mayo- Oulo Health District. The highest malaria prevalence was recorded in Bala (57%) while the lowest was observed in Mayo- Oulo (16.4%), both in the Mayo Oulo health district. Out of the total 362 children with complete data, only 102(28.2%) presented with fever (temperature ≥ 37,5°C) at the time of sample collection and were tested on RDT. Amongst these 91(89.2%; 95% CI: 82.0–94.2) were positive for malaria. Microscopic examination of all 362 children revealed 119 malaria positive cases, giving an overall prevalence of 32.9% [95% CI: 28.2–37.8]. *Plasmodium falciparum and P*. *malariae* were the parasite species found with *P*. *falciparum* representing 86.6% of single infections. There were only 8.4% single infections of *P*. *malariae* while mixed infections of *P*. *falciparum* and *P*. *malariae* represented 5%. No *Plasmodium ovale* or *Plasmodium vivax* infection was found. There were more infections in males 66(37.7%) compared to the females 53(28.3%) but the difference was not significant (p = 0.058).

Generally, based on the age groups, the prevalence was higher amongst children less than 5 years old compared to the older group. Comparing results from Microscopy, RDT and PCR amongst the age groups, only the prevalence by microscopy showed a significant difference (p = 0.023) between the two age groups. There was no observed significant differences between RDT and PCR (p>0.05) between the ˂5 and ≥5 age groups.

Parasite densities ranged from 40parasites/μL to 129600parasites/μL of blood with a geometric mean (GMPD) of 1783 ±19118parasites/μL of blood. The difference between the two age groups was not significant (P = 0.096). Of the 185 analysed by PCR, 123(66.5%; 95% CI: 62.4–75.9) were positive. PCR Analysis also confirmed 11 cases that were missed by microscopy (Table 3).

**Table 3:** Prevalence of malaria and parasite load by age group and by test procedure.

	RDT(Confirmed malaria, T°C≥37.5)		Microscopy		PCR		Parasitaemia (GM ± SD; Min-Max)	
**Age group**	**˂5**	**≥5**	**˂5**	**≥5**	**˂5**	**≥5**	**˂5**	**≥5**
**N**	58	44	182	180	99	86	86	33
**Positivity**	52	39	70	49	67	56	2224±23008 Min = 40 Max = 129600	1300±10318 Min = 80 Max = 69200
**Prevalence (%)**	89.7	88.6	38.5%	27.2%	67.7	65.1		
**Overall**	**89.2% (91)**		**32.9% (119)**		**66.5% (123)**		1783 ±19118; Min = 40; Max = 129600	
**Cramer’s V**	V = 0.016; P = 0.870		V = 0.120; P = 0.023		V = 0.027; P = 0.713		U = 1407.000; P = 0.096	

#### Prevalence of malnutrition

A total of 196 malnourished children were identified giving an overall prevalence of malnutrition of 54.1% (95% CI: 48.9–59.2). The rate of malnutrition did not differ significantly between the two age groups (P>0.05) with 101 (51.5%) being children aged ˂5 years and 95 (48.5%) aged ≥5 years.

The overall prevalence rates of stunting, underweight and wasting were 56.9%, 63.5% and 34.8% respectively, showing that most of the children were underweight. Although not significantly different (p = 0.223), the prevalence of malnutrition was higher in the Pitoa Health District 109(55.6%) than Mayo-Oulo 87(44.4%), with the highest prevalence recorded in Dourbeye (60.7%) and lowest in Mayo-Oulo (40.3%).

Malnutrition was significantly higher in males (60.6%) than in females (48.1%). Stunting was also significantly higher in males (66.9%) than females (47.6%). Underweight was more in males (65.7%) than females (61.5%) although the difference was not significant while wasting was more in females (37.9%) than males (31.4%).

The prevalence rates of severe stunting, severe underweight and severe wasting were 29.1%, 20.9% and 9.3% in the ˂5 age group and 18.3%, 10.0% and 7.8% in the ≥5 age group respectively, with males being generally more severely malnourished than females (p˂0.05) as well. Comparisons among age groups in males revealed that the prevalence for severe underweight was significantly higher (p = 0.002) in ˂5 than the ≥5. Although not significantly different (p = 0.296), severe stunting was higher in the ˂5 age group than the ≥5 while severe wasting was more in the ≥5’s than the ˂5 age group (p = 0.963). In females, severe stunting and severe underweight were significantly higher (p = 0.028) and (p = 0.003) respectively in the ˂5 age group compared to the ≥5 while severe wasting was higher in the ≥5 age group than the ˂5, but not statistically significant (p = 0.185).

#### Relationship between malaria and nutritional status

Out of the 119 children who had malaria 70(58.8%) were ˂5years old while 49(41.2%) were ≥5years. For the children infected with malaria parasite, the mean±SD z-scores for HAZ, WAZ and WHZ in the ˂5 age group were -1.580±2.100, -1.504±2.121 and -0.231±2.729 respectively while for the ≥5 age group, -1.245±1.925, -1.732±1.224 and -1.355±1.566 respectively.

Overall, malaria prevalence in malnourished children was 37.8% (n = 196), of which 67.2% were underweight, 56.3% were stunted and 41.2% were wasted. Among age groups, although not significantly different (p = 0.331), malaria prevalence in malnourished children was higher 33(67.3%) in the ≥5 age group than the ˂5 age group 41(58.6%).

#### Relationship between malaria, anaemia and nutritional Status

Anaemia was assessed by Hb levels. Children with haemoglobin levels less than 11 g/dL were considered to be anaemic in accordance with the WHO classification system [28]. Out of the 368 children who were recruited for the project, PCV was successfully obtained for 359 of them. The overall prevalence of anaemia in the study population was 74 (20.6%) at (95% CI: 16.7–25.0) being higher in males 37(21.3%) than in females 37(20.0%), although no significant difference (p>0.05) was observed. Pitoa Health District generally had a significantly higher (p = 0.007) prevalence of anaemia than Mayo-oulo, with the highest in Kirambo (32.8%) and the lowest in Dourbeye (3.7%). Mild, moderate and severe anaemia were prevalent in 29 (8.1%), 33 (9.2%) and 12 (3.3%) children respectively. Children ˂ 5years 40(22.2%) had a higher (p = 0.45) anaemia prevalence when compared to those ≥ 5 years 34(19.0%). (Table 4) In the prevalence of anaemia, no statistically significant differences (p = 0.422) were seen between malaria positive children and malaria negative children, as well as between the malnourished and normal children (p = 0.599).

**Table 4:** Prevalence of anaemia in the various categories of children by age group.

	Nutritional status				Malaria status				Age group	
	Malnourished		Normal		Malaria positive		Malaria negative			
**Age group**	**≤ 5**	**>5**	**≤ 5**	**>5**	**≤ 5**	**>5**	**≤ 5**	**>5**	**≤ 5**	**>5**
**N**	100	94	80	85	69	48	111	131	180	179
**Anaemic**	24	18	16	16	15	12	25	22	40	34
**Prevalence (%)**	24%	19.1%	20.0%	18.8%	21.7%	25.0%	22.5%	16.8%	22.2%	19.0%
**Overall**	**42 (21.6%)**		**32 (19.4%)**		**27 (23.1%)**		**47 (19.4%)**		**74 (20.6%)**	
**Cramer’s V**	V = 0.059; P = 0.412		V = 0.015; P = 0.849		V = 0.038; P = 0.680		V = 0.072; P = 0.262		V = 0.040 P = 0.450	
**Cramer’s V**	V = 0.028; P = 0.599				V = 0.042; P = 0.422					

#### Relationship between malaria, anaemia, nutritional and socio-economic status

Overall, the categorization of the development index into quintiles revealed that the majority of children in the study population belonged to the poor SES quintile, followed by the very poor. Indicators for socio-economic status included; the number of people living in a household, the level of education of caregiver and family head, employment status of family head, type of housing, type of toilet, source of lighting and cooking energy, availability of potable water and possession of household assets.

Children belonging to the average SES quintile were observed to have the highest prevalence of malaria (42.4%) as opposed to those of the rich SES quintile who had the lowest prevalence (18.0%). The overall prevalence of malnutrition was 54.1% with children from the poor SES quintile observed to have the highest prevalence of malnutrition (64.9%) while children in the very rich SES quintile had the lowest prevalence (48.0%). With regards to anaemia, overall prevalence was 20.6%, with children belonging to the poor SES quintile having the highest prevalence of anaemia (26.8%) while those in the rich level had the lowest prevalence (7.1%).

#### Predictors of malaria, malnutrition, and anaemia

For Malaria, the model demonstrated that location [OR = 1.62, (95% CI: 1.28–2.05); (p˂0.001)] and nutritional status [OR = 2.07, (95% CI: 1.22–3.53); (p = 0.007)] were significant predictors of malaria, with nutritional status having 2 times greater risk of malaria (Table 5). Other risk factors for malaria were; level of education of care giver (p˂0.001), development index (p = 0.042) and family head employment status (p˂0.001).

**Table 5:** Logistic regression model predicting risk factors of malaria.

Predictors	B	S.E.	Wald	df	Sig.	Exp(B)	95% C.I. for EXP(B)	
							Lower	Upper
Villages	.484	.121	16.001	1	.000	1.622	1.280	2.055
Religion	.378	.312	1.466	1	.226	1.460	.791	2.692
Gender of family head	1.146	.741	2.391	1	.122	3.147	.736	13.457
Family head relationship with child	.195	.121	2.622	1	.105	1.216	.960	1.540
Care giver relationship to family head	-.886	.838	1.118	1	.290	.412	.080	2.130
Marital status of family head	6.247	5424.315	.000	1	.999	516.599	.000	.
Level of education of care giver	-1.153	.318	13.189	1	.000	.316	.169	.588
Level of education of family head	.375	.335	1.253	1	.263	1.455	.754	2.808
Type of housing	-.025	.085	.086	1	.770	.975	.825	1.153
Household size	.298	.199	2.236	1	.135	1.347	.912	1.990
Gender of child	.292	.263	1.229	1	.268	1.339	.799	2.243
Development index	-.282	.138	4.154	1	.042	.755	.576	.989
Nutritional status	.729	.271	7.221	1	.007	2.073	1.218	3.528
Child age	.267	.263	1.032	1	.310	1.306	.780	2.186
Family head employment status	-.393	.084	21.906	1	.000	.675	.573	.796

The LRM also demonstrated sex of child [OR = 1.65, (95% CI: 1.05–2.59); (p = 0.029)], level of education of care giver [OR = 2.41, (95% CI: 1.39–4.16); (p = 0.002)], and malaria status [OR = 1.89, (95% CI: 1.12–3.19); (p = 0.017)] as significant predictors of malnutrition as shown above (Table 6). The level of education of the care giver [OR = 2.41, (95% CI: 1.39–4.16); (p = 0.002)] was associated with 2 fold elevated risk of being malnourished. Furthermore, the model also identified other risk factors for malnutrition like family head relationship with child (p = 0.031).

**Table 6:** Logistic regression model predicting risk factors of malnutrition.

Predictors	B	S.E.	Wald	df	Sig.	Exp(B)	95% C.I. for EXP(B)	
							Lower	Upper
Villages	-.168	.111	2.277	1	.131	.845	.680	1.051
Religion	-.291	.281	1.079	1	.299	.747	.431	1.295
Gender of familyhead	-.241	.609	.156	1	.692	.786	.238	2.594
Family head relationship with child	-.229	.106	4.668	1	.031	.796	.647	.979
Care giver relationship to family head	1.072	.622	2.974	1	.085	2.921	.864	9.877
Marital status of family head	.294	.357	.681	1	.409	1.342	.667	2.702
Level of education of care giver	.879	.278	9.963	1	.002	2.408	1.395	4.156
Level of education of family head	-.478	.289	2.735	1	.098	.620	.352	1.093
Type of housing	-.062	.077	.662	1	.416	.939	.808	1.092
Household size	.087	.178	.237	1	.627	1.090	.769	1.546
Gender of child	.502	.230	4.789	1	.029	1.652	1.054	2.591
Development index	.087	.129	.461	1	.497	1.091	.848	1.404
Anaemic status	.228	.284	.649	1	.421	1.257	.721	2.191
Malaria status	.638	.268	5.680	1	.017	1.892	1.120	3.196
Child age	-.004	.232	.000	1	.985	.996	.632	1.568
Family head employment status	.080	.070	1.293	1	.256	1.083	.944	1.243

Anaemia had family head relationship with child [OR = 1.41, (95% CI: 1.1–1.8); (p = 0.006)] and socio-economic development index [OR = 1.45, (95% CI: 1.05–2.00); (p = 0.023)] as significant risk factors (Table 7).

**Table 7:** Logistic regression model predicting risk factors of anaemia.

Predictors	B	S.E.	Wald	Df	Sig.	Exp(B)	95% C.I. for EXP(B)	
							Lower	Upper
Villages	-.043	.136	.099	1	.753	.958	.734	1.251
Religion	.555	.341	2.651	1	.103	1.742	.893	3.399
Gender of familyhead	-.196	.836	.055	1	.815	.822	.160	4.228
Family head relationship with child	.344	.126	7.431	1	.006	1.411	1.102	1.807
Care giver relationship to family head	.486	.742	.430	1	.512	1.626	.380	6.962
Marital status of family head	.050	.464	.012	1	.914	1.051	.424	2.608
Level of education of care giver	-.238	.348	.468	1	.494	.788	.399	1.558
Level of education of family head	-.038	.367	.010	1	.919	.963	.469	1.978
Type of housing	.075	.103	.528	1	.467	1.078	.881	1.319
Household size	-.100	.223	.200	1	.654	.905	.584	1.402
Gender of child	-.052	.283	.034	1	.854	.949	.545	1.652
Development index	.373	.164	5.173	1	.023	1.452	1.053	2.001
Malaria status	.233	.287	.660	1	.416	1.262	.720	2.215
Child age	.363	.324	1.253	1	.263	1.438	.761	2.714
Family head employment status	.344	.280	1.507	1	.220	1.411	.814	2.444

## Discussion

Malaria represents a major public health problem. The overall prevalence of malaria in the study sites was 32.9%. The prevalence of malaria was also observed to be significantly higher (p<0.01) in Mayo- Oulo health district (38.5%) than Pitoa health district (27.6%), though both were peri-urban settings, probably because our study covered only 6 villages as opposed to earlier studies which covered a wider range of villages. This result was lower than 44.3% observed by Kimbi et al., in the Mount Cameroon region [29], and could be linked to the recent scale-up of intervention strategies such as the free treatment for malaria in children and the use of LLIN’s (Long Lasting Insecticide-treated Nets) by the National Malaria Control Program (NMCP)[30].

Also, our findings revealed that the prevalence of malaria was inversely related to age. Such increased susceptibility of younger children to infections has been attributed to their poorly developed immune systems [31].

The logistic regression analysis demonstrated that location [OR = 1.62, (95% CI: 1.28–2.05); (p˂0.001)] and nutritional status [OR = 2.073, (95% CI: 1.22–3.53); (p = 0.007)] were significant risk factors for malaria. Thus there are 1.62 times more chances that a child will become infected with malaria in Bala than in Mayo-Oulo. The key to effective management of malaria is prompt and accurate diagnosis. The WHO recommends that malaria case management where possible should be based on parasitological diagnosis, except when considering young children in endemic areas where lack of resources or urgency of response temporarily limits its application [32, 33]. Malaria control in Cameroon relies principally on anti-vector intervention using long lasting insecticide nets (LLIN) [2] and mainly through large scale campaign and free distribution of the nets. Therefore the Ministry of public health of Cameroon should continue to implement serious plans to rapidly scale-up control measures against the parasite nation-wide, to further reduce malaria infection to the barest minimum and possibly eradicate it, thereby lessening the socio-economic burden.

With regards to malnutrition, the overall prevalence was 54.1% with an overall prevalence of 56.9% for stunting. Although this is higher than is reported previously in the same region with 40.2% for malnutrition and 37.7% for stunting, [9]. It is similar to those reported by Nkuo-Akenji et al., [34] in the Mount Cameroon area. However, the high malnutrition prevalence observed in our study is in conformity with earlier reports from Africa in general, specifically in sub-Saharan Africa whose prevalence stands at 55.2% for stunting as reported by Kwena et al., 2014. The high stunting prevalence equally underscores the fact that, chronic malnutrition is still a heavy burden in the area, which confirms earlier findings by Ngwa Akonwi et al., 2015. Stunting is generally associated with long term undernutrition whereas wasting is a manifestation of recent and acute undernutrition [35]. The prevalence of malnutrition shows about half of the children in our study (196) to be malnourished which is perfectly in line with the WHO report which states that fully half of the human family, some 3 billion people, suffer from malnutrition of one kind or another. [36]

With regards to malnutrition, the high prevalence is indicative of poor feeding habits, absence of health/ nutritional education and lack of proper knowledge on nutrition and balanced diets as well as the low level of education of the caregivers in the studied area. The observed results on the prevalences of stunting, underweight and wasting in both males and females, was consistent with the observations of Sumbele et al.,[37] in the Mount Cameroon area, Wamani et al., in Tanzania that stunting and underweight were common among males than females in all age groups [38, 39].

A higher prevalence in malnutrition was observed among the ˂5years (55.5%). This is unlike reports from South Western Ethiopia where older children were more likely to suffer from undernutrition. This difference is probably because younger children are more vulnerable to infection due to their poorly developed immune systems [31]. Poor feeding habits may also have contributed to the higher prevalence in the ˂5 age group in the study.

Logistic regression analysis was run and it identified malaria status, sex of child and level of education of caregiver as significant predicting risk factors for malnutrition. As revealed by the odd ratios, the level of education of the care giver was associated with 2 fold elevated risk of being malnourished. This implied that children of illiterate mothers were 2.40 times more likely to become malnourished compared to those that had basic education.

Unlike Snow et al., [22] who reported no effect of anthropometric indices on susceptibility to malaria in The Gambia, our results showed an association between malaria and malnutrition. Ehrhardt et al., [1] also identified malnutrition as a major underlying cause of malaria. The relationship between malaria infection and nutritional status was a two-way association. Malnourished children were 2.07 times more likely to become infected by malaria parasite compared to well-nourished children. On the other hand, malaria positive children were 1.89 times more likely to become malnourished than those uninfected. This implies that on one hand, malaria may cause malnutrition, whereas on the other hand, malnutrition may exacerbate the disease. This confirms the controversial synergistic relationship between malaria and malnutrition as previously reported elsewhere [14]. Overall, while children who were stunted and underweight had a higher prevalence of malaria parasite, those wasted had a lower prevalence.

The foremost aim of nutritional assessment studies is to determine the actual magnitude of under nutrition and thereby introduce appropriate nutritional intervention programmes to improve the existing nutrition situation [40**–**42].

In order to meet up with the World Health Assembly’s Global Nutrition Targets 1 and 2 for 2025 on a 40% reduction in the number of children under-5 who are stunted as well as reduce and maintain childhood wasting to less than 5%, then effective strategies need to be sought [43].

Therefore, appropriate measures such as improved nutritional practices could be recommended in areas with high malnutrition [29]. Another way forward would be to expand the health benefits and improve control strategies from the Ministry of Public health for rapid scale-up of already proven nutrition-specific and nutrition-sensitive interventions for the vulnerable malnourished children in the country.

Anaemia is widely distributed and often found in developing countries [44]. Chronic anaemia during childhood is associated with retardation in physical development, cognition and school performance [45], while severe anaemia (haemoglobin ˂ 7g/dL) is responsible for more than half of the deaths attributed to malaria in children under five years of age.

Anaemia is a key public health challenge in Cameroon. Its prevalence as revealed by our study was 20.6%. This is unexpected in a peri-urban community where malaria is hyper endemic. Earlier studies by Jourdan et al., reported prevalence as high as 82% for children attending a clinic in Northern Cameroon [46]. The prevalence of anaemia in our study is lower than 22.6% reported in Brazil in 2011 [47], lower than the 62% recently recorded in under 12 children in Odisha, India in 2016 [48], and the 29% recorded in the Demographic Health Survey (DHS) in Honduras in 2011 amongst 6-59months old [49]. It is also lower than the most recent data (61%) reported by the WHO in 2017 [7].

In our study, the lower prevalence of anaemia observed may probably be due to the low malaria prevalence in the studied area coupled to the fact that ours was a community-based household survey and most of the children examined were healthy children and not necessarily sick children in a hospital. It also revealed mild moderate and severe anaemia to be 8.1%, 9.2% and 3.3% respectively. This indicates that severe anaemia was rare in the area. Meanwhile the WHO prevalence rates for mild, moderate and severe anaemia stood at 25%, 33% and 3% in 2017.

Anthropometry has become a practical tool for evaluating the nutritional status of populations, particularly of children in developing countries and nutritional status is the best indicator of the global well-being of children [41]. Despite the fact that our study on nutritional status was limited to anthropometry, that iron deficiency adversely affects human health is widely recognized. Anaemia could be caused by iron deficiency as a result of; a decrease in haemoglobin concentration; Even though malaria causes chronic anaemia, impaired growth and delayed development in young children [11], in this study, no significant associations were found between anaemia and malaria (p = 0.422). Although no significant differences were observed in the prevalence of anaemia between the different sexes, anaemia was higher in males than in females. Similar observations were made in Tanzania [39]. This may be explained by the fact that males were malnourished than females (p = 0.018).

The results equally showed that there was no statistically significant association between anaemia and malnutrition (p = 0.599). Contrary to our findings, malnutrition was reported in Ghana [1], to be a fundamental factor contributing to malaria-associated morbidity and anaemia, even if the latter exhibits multifactorial patterns. Most findings (in Malaysia and Western Kenya) [21, 50] also reported correlation between education level and anaemia. But our study failed to find such an association.

The LR analysis significantly identified development index and family head relationship with child as significant risk factors for anaemia. Meaning, anaemia correlated with socio economic status (p = 0.023). Children with a low SES were 1.45 times more likely to become anaemic [OR = 1.45, (95% CI: 1.05–2.0)] compared to children in the high socio-economic level.

All in all the study highlighted the fact that, malaria and malnutrition may not have been the causes of anaemia in the study population. Our findings show no linear relationship between anaemia and malaria or malnutrition, suggesting the possible involvement of other factors in the development of anaemia in children. Therefore, the results are suggestive of the fact that the anaemia observed in the children may have been due to other presumptive causes like helminthes among others. The prevention as well as timely management of anaemia is essential to attain Sustainable Development Goal- 3 (SDG) on ensuring healthy lives and promoting well-being for all at all ages. Further actions are required to reach the World Health Assembly target of a 50% reduction of anaemia in women of reproductive age by 2025 [36].

Socio-economic status has been defined as a position that an individual or family occupies with reference to prevailing standards of cultural and material possessions, income and education [51]. Our findings revealed that majority of children in the study population belonged to the poor SES quintile, which is not surprising, considering the fact that the community being studied has a low standard of living and confirms previous studies in the same area by Akonwi Ngwa et al., 2015. Socioeconomic factors may influence malaria morbidity, although their respective roles are somewhat controversial [51]. The findings on SES are in support of the fact that, anaemia was significantly associated (p = 0.023) to socio-economic status and is in line with earlier findings which state that low economic status, less education, and poor health of mothers due to meagre dietary intake are the main causes of anaemia [48]. Child undernutrition is also influenced by several socio-economic and socio-demographic variables (e.g., sex, age, birth order, education and occupation). This too was seen in our study but we did not find statistically significant association between malnutrition and development index (p = 0.348). Socio-economic status did not influence malnutrition.

## Conclusion

Control and intervention programmes will have limited effects if we fail to recognize and consequently target the underlying causes of the diseases. Nutritional counselling and health education interventions of mothers followed by prompt feeding programs have to specifically focus on improving the health of the malnourished and anaemic. These, alongside continuous proper malaria-control measures of proven efficacy, may be apt to reduce the burden of malaria, anaemia, malnutrition and poverty on a large scale. This study therefore elucidates the interplay between the various health determinants and provides preliminary baseline data needed by policy makers, programme planners and implementers, non-governmental organizations, for strategic planning and implementation of the rapid scale-up of nutritional interventions as well as scaled-up malaria parasite control measures in the north region of Cameroon.

## Recommendations

Children screened for malaria should also be treated for anaemia and helminthes, especially in the poor communities. Children with pale mucosae should be provided with iron supplementation. There is need for appropriate nutritional and deworming interventions so as to ameliorate the anaemic status of the children. Further research needs to be done to ascertain the exact cause of the anaemia in the children.
